# Reporting all results efficiently: A RARE proposal to open up the file drawer

**DOI:** 10.1073/pnas.2106178118

**Published:** 2021-12-21

**Authors:** David D. Laitin, Edward Miguel, Ala’ Alrababa’h, Aleksandar Bogdanoski, Sean Grant, Katherine Hoeberling, Cecilia Hyunjung Mo, Don A. Moore, Simine Vazire, Jeremy Weinstein, Scott Williamson

**Affiliations:** ^a^Department of Political Science, Stanford University, Stanford, CA 94305;; ^b^Immigration Policy Laboratory, Stanford University, Stanford, CA 94305;; ^c^Department of Economics, University of California, Berkeley, CA 94720;; ^d^National Bureau of Economic Research, Cambridge, MA 02138;; ^e^Center for Effective Global Action, University of California, Berkeley, CA 94720;; ^f^Immigration Policy Laboratory, ETH Zurich, 8092 Zurich, Switzerland;; ^g^Center for Comparative and International Studies, ETH Zurich, 8092 Zurich, Switzerland;; ^h^Richard M. Fairbanks School of Public Health, Indiana University, Indianapolis, IN 46202;; ^i^The Charles and Louise Travers Department of Political Science, University of California, Berkeley, CA 94720;; ^j^Haas School of Business, University of California, Berkeley, CA 94720;; ^k^Melbourne School of Psychological Sciences, University of Melbourne, Melbourne, VIC 3010, Australia;; ^l^Department of Social and Political Sciences, Bocconi University, 20100 Milano, Italy

**Keywords:** research transparency, registries, null findings, publication bias, file drawer problem

## Abstract

While the social sciences have made impressive progress in adopting transparent research practices that facilitate verification, replication, and reuse of materials, the problem of publication bias persists. Bias on the part of peer reviewers and journal editors, as well as the use of outdated research practices by authors, continues to skew literature toward statistically significant effects, many of which may be false positives. To mitigate this bias, we propose a framework to enable authors to report all results efficiently (RARE), with an initial focus on experimental and other prospective empirical social science research that utilizes public study registries. This framework depicts an integrated system that leverages the capacities of existing infrastructure in the form of public registries, institutional review boards, journals, and granting agencies, as well as investigators themselves, to efficiently incentivize full reporting and thereby, improve confidence in social science findings. In addition to increasing access to the results of scientific endeavors, a well-coordinated research ecosystem can prevent scholars from wasting time investigating the same questions in ways that have not worked in the past and reduce wasted funds on the part of granting agencies.

The social sciences have made impressive progress in adopting transparent research practices that facilitate verification and replication. Study registration in publicly available registries and data sharing are increasingly common (1, 2). Norms of opacity persist, however, around the (non)reporting of null results. These norms effectively skew the available evidence toward statistically significant effects (i.e., results that confirm theory or treatment effectiveness), even if those effects are actually false positives that will not replicate (3). Researchers are also incentivized to produce novel or significant effects, which can lead them to use questionable practices, making results seem more newsworthy. If follow-up studies reveal null results that cannot readily find a publication outlet, false positives will not be corrected in the scientific literature.

To mitigate these problems, we propose a framework to report all results efficiently (RARE). This proposed framework resulted from an NSF-supported workshop that convened scholars from diverse disciplines, compliance officers, and funders to discuss the failures of multiple stakeholders to resolve the “file drawer problem” (4). In supporting this workshop, NSF senior staff made clear that change in their procedures is best fostered through a reform consensus in the scientific community and that the workshop should be designed to seed such a consensus.

Our workshop consensus was that full reports to the scientific community of results, whether or not initial hypotheses were confirmed, required an integrated set of procedures to sustain a new norm of full reporting. Our core aim is the full reporting of results on existent but underutilized public registries. RARE should, therefore, be seen as a framework of reforms in which changed incentives coming from each element of the scientific community will sustain a new equilibrium of full reporting in social science practice, with a focus on experimental and other empirical research in which hypotheses are publicly registered.

To be sure, RARE will require a reestablishment of current scientific norms around research planning, reporting, and citation. Our proposal asks that several actors and institutions—researchers, donors, institutional review boards (IRBs), registries, and journals—make meaningful changes to their workflows, which may require onerous work up front. It is our hope, however, that such effort will save time and resources in the long run, both for researchers who adopt the practices we recommend and for the scientific community as a whole. Indeed, there is growing evidence that, after openness is integrated into researcher workflows, a significant amount of time can be saved when updating or building on past work (5, 6). Indeed, the “efficiently” in our framework’s acronym refers both to the costs saved to individual scholars with newly designed registries for reporting all results and to the research community with easy to access unpublished research findings. This can be especially advantageous to scholars outside of leading academic networks who are less likely to be apprised (via informal channels) of null results. Implemented in full and broadly bought into, RARE also offers new rewards and incentives for transparency that are unnecessarily challenging under the current paradigm.

## Wasted Funding and Missed Opportunities

Null results—those findings that do not provide significant support for a proposed theory or experimental treatment—remain largely hidden. According to one assessment, 50 to 80% of Food and Drug Administration (FDA)–and NIH-funded projects do not result in publication (7). Null results produce a particularly dismal publication rate; fewer than a quarter of null findings from the NSF-funded Time-Sharing Experiments in the Social Sciences studies were published, while nearly two-thirds of those with all or most hypotheses reporting statistically significant results were published (8). The near disappearance of null results represents an alarming degree of waste—of both scientific funding and researcher effort—and threatens the credibility of scientific inquiry. Null results carry valuable information and consequently, have potentially major implications for science and policy.

Our framework speaks directly to all social science research where, under current norms, preanalysis plans (PAPs) are an expected element of the scientific workflow. As an initial target, the approach can be easily applied to experimental research, where PAPs are already common. Going forward, we believe the framework can be applied to all hypothesis-testing research. Other types of research (e.g., exploratory research, qualitative research, agent-based modeling, and formal theory) face different institutional constraints on transparency; we leave for future consideration whether and how RARE can be adapted to the systems supporting these kinds of research. From a Bayesian and meta-analytic perspective, all results are consequential for updating effect size estimates (9). In fact, a well-designed and well-powered study with a precisely estimated null may be more informative in some cases than a statistically significant estimate. Suppression of null results impedes researchers’ abilities to learn when effects do not exist, increasing the risk that a false-positive result, once published, could inspire an endless string of failed attempts at replication and fruitless extensions.

An obverse problem may also exist. Replications confirming previous findings may not be newsworthy enough to merit publication, even when they feature new data and additional robustness tests. Two recent studies suggest that replications that overturn findings are published more easily (10, 11). If this is the case, many confirming studies may live in file drawers, while disconfirming studies enter the scientific domain. Other self-reported survey evidence shows that many researchers have found success in publishing successful replications, while others have not (12). Either way, our understanding of the replicability of the majority of published research is incomplete, presenting risks to our confidence in published findings—robust or otherwise.

Locked file drawers also have implications for research subjects who choose to participate in the expectation that science will provide public benefits. To the extent that the data they generate are hidden, participants themselves may have been misled, and core ethical principles for research involving human subjects and participants, as stipulated in the guiding Belmont Report, may be threatened (13).

Yet, signs of remedial action exist. Over 5,000 journals and organizations have signed onto the Transparency and Openness Promotion guidelines (14), pledging greater disclosure of the scientific process. Dissemination of preprints (sometimes called working papers) by social scientists has also grown dramatically in recent years—over 30,000 representing 21 disciplines have been shared on the Open Science Framework servers alone (15). Additionally, journals are accelerating the dissemination of scientific knowledge in public health emergencies through rapid data publication and by sharing preprints (16). New meta-analytic techniques integrate unpublished research outputs discovered by trawling trial registries and regulatory agency websites or analyzing unpublished data (17).

Most importantly, social science registries in which researchers deposit their PAPs and report on the progress of their projects are becoming more accessible. (The American Economic Association [AEA] RCT [randomized, controlled trial] Registry, the Evidence in Governance and Politics Registry, and the Open Science Framework Registries are prominent examples.) However, significant hurdles remain. For one, few registries make it clear how researchers should report results to accompany their PAPs. Second, they do not provide fields for interpretation of these results that could guide future researchers. Third, registries without fields for the reporting of findings add to the costs of replication by other scholars. It is no wonder that reporting rates, especially of null findings, are low. Based on our analysis of the AEA Registry metadata posted in August 2021 (18, 19), of the 3,845 registered projects that had passed their stated end date, only 41.6% (1,598) had reported completion and only 10.9% (421) reported results. In a random selection of 30 of these reports that had provided results, null results were mentioned in nearly all; however, among those studies that had reported at least one null finding, a null result could be linked to a prespecified hypothesis in only 17%. A similar result was found by Alrabaha’h et al. (20). This stark gap in reporting results was the motivating observation leading to the proposed RARE framework. Second, registries do not distinguish results that ultimately confirm hypotheses from those that do not, nor do they provide fields for interpretation of these results. Finally, registries without fields for the reporting of findings add to the costs of replication by other scholars. RARE is our proposal to overcome these limitations.

## A RARE Framework to Reporting All Results Efficiently

Effective solutions to the file drawer problem should build on existing infrastructure. The key innovation of the RARE framework is to link the elements of that infrastructure in a way that encourages authors to report the results of all their hypotheses in an existing study registry and to do so at low cost to their research programs.

**Fig. 1.**
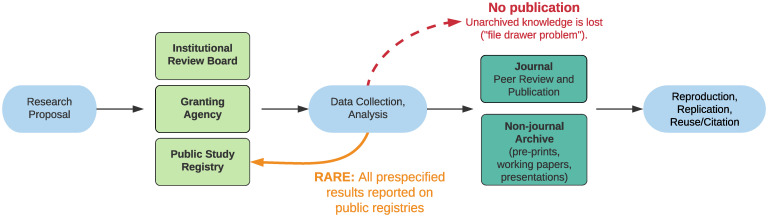
The importance of RARE. This visualization of the scientific ecosystem shows leverage points where interventions are possible. Arrows indicate the flow of a study or research project. The thick orange arrow indicates the keystone of our framework for posting all results on public registries. The dashed red arrow indicates where knowledge is at risk of being lost due to current deficiencies.

We believe existing processes present multiple opportunities for improved transparency along these lines, including actions by investigators themselves, study registries, IRBs or ethics review committees, granting agencies, and journals. Fig. 1 contains a schematic representation of this ecosystem and the roles played by different stakeholders.

Our proposal links, in a common framework, five units in the scientific ecosystem. Here, we describe actions and policies that can support the emergence of a new full-reporting norm. We envision our proposed actions and policies initially as voluntary but with the expectation that over time, new norms will emerge in social scientific subdisciplines that will create bottom-up enforcement of these norms.

### Registries.

Registries are the keystone institution for RARE, but we propose several modifications. First, while they now have a robust template for posting PAPs and research designs, most registries need to add fields for reporting on findings as does clinicaltrials.gov. Second, to be more useful for future replication, registries should also include open-ended fields where investigators can suggest interpretations of these findings. While this is important for positive and null findings alike, the RARE framework provides enhanced standards for reporting of null results, which too often remain unpublished. For instance, future researchers would want to know whether a null result was due to an underpowered setup, a glitch in the protocol, or a likely theoretical failure. Third, as today’s filings vary in quality and completeness, some standardization in reporting should be encouraged to help reduce investigator costs of posting results and give future scholars clues as to what might be improved for a replication exercise. As one possible model, Stanford’s Immigration Policy Laboratory has proposed a reporting template that would allow for full disclosure at relatively low cost to the researchers and could be a model for registering both experimental and observational studies (20). Fourth, given that some journals restrict authors from publishing manuscripts on personal websites or preprint servers prior to publication, registries might follow the example of clinicaltrials.gov and allow investigators to embargo the results section of their registries for a period of time. Finally, registries that host these reports could enhance their discoverability and usefulness by making their contents findable in search engines like Google Scholar and by providing citable digital object identifiers. This last modification can help create incentives for researchers who stand to benefit from increased visibility and citation of their work. Indeed, for less established scholars, work posted in registries might serve as useful indicators of research productivity beyond conventional publication metrics.

### Investigators.

If RARE were adopted, investigators would outline a plan in their proposals to both IRBs and funding agencies for how results will be reported even if they are not published through the peer-reviewed journal process, indicating where studies will be registered and when applicable, how subjects will be assured that all results will be made publicly available. They should also be expected to provide access to registrations and results from previous grants upon request.

In practice, this would mean researchers would need to preregister their hypotheses in a public registry, include a link to their registration in grant proposals and IRB protocols, and deposit a summary of their results on that same registration within a certain period of time, perhaps depending on funder or registry policy or disciplinary convention. While detailed PAPs can help researchers think through and elicit feedback on their research designs, study registrations as we propose under RARE do not necessarily need to take this form. Rather, they need only be detailed enough for investigators to indicate the results of preplanned hypotheses. These are not excessive burdens. A recent survey of social scientists indicates that writing a PAP shifts time costs to the beginning of a project’s life cycle, rather than adding to it, while often improving the project design overall, and thus, there is no loss in research efficiency (21).

### IRBs and Ethics Review Committees.

IRBs and ethics review committees oversee research involving human subjects and participants, but to our knowledge, they have not played a meaningful role in plans for full reporting. These boards and committees are charged with upholding the principle of beneficence. Consistent with this charge and with a recent proposal to incorporate IRBs into the open science movement, IRBs and ethics review committees need to be assured that researchers will share results from studies with their participants and human subjects (22). Approval should be contingent on a plan to fulfill commitments to subjects that their participation will have the promised social benefit of contributing to knowledge.

### Granting Agencies.

Granting agencies typically ask applicants to list publications from previous grants. We propose they also ask grantees to identify registrations from previous grants and confirm whether all results have been disseminated through reports posted on public study registries. We are not prescribing any standard for these reports; this may vary by research community and granting agency. We hope, however, that this requirement will incentivize peer reviewers to pay attention to the transparency of previous research and thereby, incentivize researchers to update registrations before they submit future proposals.

There may also be a need for granting agencies in the social sciences to rethink how they support human infrastructure or compliance offices, commonly employed at medical research institutions. To the extent that registration fulfillment reports are best suited for project managers, research assistants, and other laboratory-supporting personnel, granting agencies for the social sciences should consider supporting those personnel in addition to specific projects. This support may be particularly important to researchers at less well-funded institutions, ensuring they have the resources to comply with evolving norms and policies.

Of course, funding for social science research is limited, much more so than for biomedical research. If granting agencies are to efficiently distribute funds to worthwhile projects that will result in useful evidence, a shift will be needed in how funders value results and the effort required to share them. Our hope is that as file drawers open and more credible bodies of evidence become accessible, the improved reputation of social science will justify greater public and foundation allocations for social science research.

Major research funders, such as the Gates Foundation, have already begun requiring full reporting of results, but their criteria remain somewhat unclear, and results of registered hypotheses are not required to be made publicly available. Relatedly, NIH and FDA policies allow them to levy fines on institutions that do not report full results of funded trials. Yet, to date no noncompliant institutions have been fined to our knowledge, and these agencies have foregone several billion dollars that might fund other activities (23). In our view, these reporting policies should be implemented more strictly. Likewise, if the research community supports the proposed RARE framework, the NSF could then institute and enforce similar policies—to start at least for experimental and other hypothesis-testing research—to change norms regarding full reporting.

### Journal Editors and Peer Reviewers.

Authors could similarly be incentivized by journal editors and peer reviewers to verify that all results from their preanalysis plans or hypotheses have been publicly reported in a study registry (or at least posted subject to an embargo period). For example, editors could announce, contingent on disciplinary norms as reflected in the reports of peer reviewers, that such reporting will be a factor in their decision to publish. (To be sure, the requirements are less relevant for other types of scholars, such as formal modelers or those engaged in purely descriptive analyses.) In recent years, journals have shown they can play a key role in driving the adoption of other open science research practices. We are therefore optimistic about norm change around null results reporting if journals and other key academic gatekeepers lead the charge. Journals and other gatekeepers can reinforce incentives for open reporting by selecting research on the basis of good questions and designs rather than whether results are null or significant. There is a recent precedent for this: Blanco-Perez and Brodeur (24) show that a coordinated editorial statement led to a significant increase in the publication of null results in leading health economics journals. Other social science journals could usefully follow suit. Also impressive, since 2013 over 200 journals have accepted and published “Registered Reports” or employed “results-blind review” (25). Null and unexpected findings appear at higher rates in these published registered reports than in the traditional literature, suggesting it may be an effective mechanism for reducing publication bias (26, 27). Relatedly, authors submitting papers involving RCTs to journals of the AEA must also submit a registration number, although they are not currently required to report on all prespecified hypotheses. The RARE framework could broaden these requirements to include reporting all results before article publication.

Through this set of modifications of key components in the process of scientific production, we believe our suggestions would 1) strengthen the norm for reporting all results, including null results, in the social sciences; 2) do so without eating up valuable time for investigators who want to move on from completed research activities that may or may not be publishable in peer-reviewed journals; 3) prevent the research community from unnecessarily repeating previously conducted work due to the greater public visibility of prior results; and 4) improve the balance of evidence for meta-analyses and systematic reviews. In all likelihood, however, the tools and practices laid out in this framework will need to be adapted to meet the diverse interests and capacities of different stakeholders, disciplines, and contexts. An iterative and participatory design process could facilitate continuous improvement and effective implementation.

## Concerns, Limitations, and Costs

As with all reform proposals, RARE raises several concerns. One concern is that imposition of a null results–reporting mandate would set up a hard-edged punishment regime. Instead, the gradual emergence of norms represents a better path. To give an example of norm change, a short generation ago posting data for published articles was voluntary and rare. As norms have changed in some social science disciplines, it is now mandated with penalties (including withholding acceptance of manuscripts at many journals). We expect that the nudges we propose will ultimately yield normative changes in practice, making RARE a de facto disciplinary mandate in some social scientific subfields.

This is related to the crucial issue of career incentives for researchers. Rather than emphasizing the opportunity and reputation costs of posting null results, we offer a more optimistic view that emphasizes rewards. It is equally valid to frame future funding for those who meet transparency standards as a reward rather than lack of funding as a punishment. We can also do more as a research community. As one example, several journals currently offer awards to peer reviewers who (at high cost to themselves in terms of time) deliver high-quality reviews of submitted papers. These awards are sometimes referenced in tenure letters and highlighted in resumés. Perhaps organizations that manage leading registries such as the AEA, the Center for Open Science, or Evidence on Governance and Politics or groups such as the Berkeley Initiative for Transparency in the Social Sciences can offer public acknowledgment of high-quality reporting of preregistered results to generate similar positive incentives for scholars to adopt this practice, which generates value for the research community as a whole. Relatedly, as highlighted above, supportive infrastructure—in the form of enhanced registry fields, guidance, and improved organizational staffing to ensure investigators meet reporting needs in a timely fashion—will likely be critical for enabling these new practices.

There is also the concern that RARE prescriptions will crowd out from social science journals exciting exploratory work that had not been registered. However, this is unlikely. First, there is no evidence that exploratory results are disappearing from leading journals. The vast majority (over 80%) of published empirical work in economics, for instance, is not experimental, some of which is exploratory and not appropriate for PAPs (28, 29). Second, in some fields, what has been called “fishing” has been practiced for a long time and has yielded powerful descriptive results that were later subjected to confirmatory analyses. We are not trying to shut down this type of work, and are not proposing researchers report every single conjecture that they examined in the course of learning from their data; we suggest only that researchers should report what they said they were going to test. There is also new evidence that papers generated from preregistered analysis are likely to be published (in higher-ranked journals) and more highly cited than other work, which contradicts the view that preregistration will stifle creativity and lead to boring papers that are unattractive to the scholarly community (30). A recent review argues that registered reports show little difference in novelty and creativity but improvements in rigor and quality when compared with nonregistered reports (31).

Another concern is the time a RARE regime will cost researchers, especially early-career researchers seeking to advance their research programs with significant findings. Under a RARE regime, they may need to divert their efforts to report what could be perceived as banal findings in unpublished reports. We have a different perspective. Researchers only register hypotheses they think will be important; therefore, the results of these hypotheses, null or otherwise, are also of interest. Furthermore, we also expect that these registered nonresults will get noticed and cited, and it would, therefore, be incentive compatible for young researchers seeking recognition to disseminate them. This is the case today with the relatively new norm of data sharing in several social science disciplines, where scholars get cited for compiling datasets even if they have not generated novel findings from them. In the Comparative Politics section of the American Political Science Association, an annual prize is awarded to the best new dataset irrespective of whether the scholar who created it produced significant papers relying on it. Similar recognition could be granted to scholars who provide complete reporting of prespecified results, whether null or not.

On the issue of who should pay the cost of reporting null results, one may ask if the present practice of sharing data along with registered preanalysis plans is enough. Here, future replicators in the scientific community would collectively share the burden of unveiling failed attempts to verify theoretical conjectures. However, having the original authors produce an initial set of results is likely to be a more time-efficient approach from the point of view of the scientific community as a whole. Tasking other researchers (including possibly graduate students) with trying to reanalyze data might lead to less progress than the original study team posting results from data with which they are already very familiar (especially if basic computational reproducibility is not a given).

We are all in favor of the scientific community—and especially of graduate students in their apprenticeship years—connecting PAPs to open data that had remained unreported. However, this is not an either/or proposition; registration and complete reporting of results and community oversight reinforce each other to support the integrity of science. Indeed, we have seen a generation of data sharing in the social sciences, but still, the file drawer problem unfortunately persists (24, 32–36)

## Conclusion

Our RARE proposal would constitute an important step in achieving full transparency of the scientific process, where quality is assessed based on the questions that scholars ask, the research designs they carry out, and the theoretical informativeness of the results they generate, regardless of statistical significance or perception of newsworthiness.

If the scientific community adopted the practices we suggest, the RARE norm of posting all results of hypothesis tests on public study registries would become commonplace. The combined efforts of various actors and institutions could nudge researchers to register, update, and RARE, thereby addressing a fundamental problem for scientific advancement in a minimally intrusive manner. Full reports on registries would allow scientists to better understand the ratio of confirmed to null results following preregistered and prespecified studies and thus, also have greater confidence in the standing of current empirical facts and theory.

Furthermore, nonacademic consumers of science—policy makers, analysts, journalists, practitioners, and the public—are the ultimate beneficiaries of improved access to the full spectrum of scientific knowledge with reduced interference from publisher or researcher bias. Were this framework adopted, one could imagine a world in which less research funding is wasted, policy decisions are based on the most complete and unbiased evidence possible, and scientific expertise is more widely valued and trusted by the public.

## Supplementary Material

Supplementary File

## Data Availability

All data have been deposited in Harvard Dataverse and are indefinitely publicly available (18, 19). All analysis code can be found in GitHub (https://github.com/BITSS/RARE). All other data are included in the manuscript and/or *SI Appendix*.
