# Supplementary material for: Reporting all results efficiently: A RARE proposal to open up the file drawer
Source: Proc Natl Acad Sci U S A. 2021 Dec 21;118(52):e2106178118. doi: 10.1073/pnas.2106178118 (PMC8719896; doi:10.1073/pnas.2106178118)
Supplement: Supplementary File [file pnas.2106178118.sapp.pdf]

Supplementary Information for

Reporting All Results Efficiently: A RARE proposal to open up the file drawer

David D. Laitin, Edward Miguel, Ala' Alrababa'h, Aleksandar Bogdanoski, Sean Grant, Katherine Hoeberling,Cecilia Hyunjung Mo, Don A. Moore, Simine Vazire, Jeremy Weinstein and Scott Williamson

David D. Laitin.
E-mail: dlaitin@stanford.edu

This PDF file includes:

Supplementary text

Supporting Information Text

Methods. We downloaded "Registrations in the AEA RCT Registry (2013-05-15 through 2021-07-31)" as a .csv file from https://dataverse.harvard.edu/dataset.xhtml?persistentId=doi:10.7910/DVN/NOQ7E3. We then randomly selected 30 completed trials that had passed their stated end date and provided results in a journal publication, working paper, or other report. Our randomization code can be found with Dataset S1 on the Harvard Dataverse (see access instructions below).

We manually searched the registry for each of these 30 trials using IDs found in the dataset provided by the Registry. In the "Post-Trial" section of each registration, we accessed the paper or report linked under "Reports, Papers, and Other Materials". If the link on the registry was broken, we searched the internet for the same paper. The trial IDs, URLs, and report links used for this analysis can be found in Dataset S1.

We searched these documents for these terms: "significant", "significance level", "null", and "p-value". We also checked the provided tables for stars (*) denoting statistical significance levels.

If the trial was post-registered (i.e., registered after the end of the intervention as stated in the registration), we did not count any reported nulls as pre-specified even if the registration or pre-analysis plan identified hypotheses connected to those results. "N/A" in Dataset S1 indicates these trials were post-registered.

For pre-registered trials (i.e., those whose registrations were added to the registry prior to the stated end of an intervention), we counted papers that reported test null results at all, as well as whether any of those reported results were connected to pre-specified hypotheses in the trial registration or pre-analysis plan.

The results of this analysis can be found in Dataset S1.

Materials. R script used to generate the random list of 30 trials can be found at https://github.com/BITSS/RARE.

Dataset S1. Analysis data can be found on the Harvard Dataverse (DOI: https://doi.org/10.7910/DVN/4F0BZL).
